# Twin growth discordance trajectories and fetal growth velocity for predicting adverse neonatal outcomes: a multicenter cohort study

**DOI:** 10.3389/fmed.2026.1851509

**Published:** 2026-06-17

**Authors:** Minhuan Lin, Xuewen Huang, Yuheng Zhou, Shuting Xia, Dongmei Duan, Yingnan Ye, Yiqing Chen, Linhuan Huang, Jiying Wen, Yanmin Luo

**Affiliations:** 1 Department of Obstetrics & Gynecology, The First Affiliated Hospital of Sun Yat-Sen University, Guangzhou, China; 2 Guangdong Provincial Clinical Research Center for Obstetrical and Gynecological Diseases, Guangzhou, China; 3 Department of Obstetrics & Gynecology, Zhongshan City People's Hospital, Zhongshan, China; 4 Guangdong Women and Children Hospital, Guangzhou, China

**Keywords:** fetal growth velocity, growth discordance, longitudinal trajectory, prenatal ultrasound, twin pregnancy

## Abstract

**Background:**

Twin pregnancies are associated with a substantially increased risk of adverse neonatal outcomes. However, it remains unclear whether longitudinal changes in intertwin growth difference and fetal growth rate add prognostic information.

**Objective:**

To characterize longitudinal patterns of intertwin growth discordance and evaluate whether combining these patterns with fetal growth velocity (FGV) improves prediction of adverse neonatal outcomes in twin pregnancies.

**Methods:**

This multicenter retrospective cohort study included 1,930 twin pregnancies with at least three ultrasound examinations, with one center used for model development and another for external validation. Group-based trajectory modeling (GBTM) was applied to identify longitudinal discordance patterns. FGV was calculated by linear regression of serial estimated fetal weight. Multivariable logistic regression models were constructed to evaluate predictive performance, with exploratory machine learning analysis performed to assess potential non-linear effects. Model discrimination was evaluated using area under the receiver operating characteristic curve (AUC).

**Results:**

Four intertwin growth discordance trajectories were identified: Stable low (84.1%), Low rapid increasing (8.5%), High decreasing (5.1%), and High increasing (2.3%). Compared with the Stable low trajectory, both High decreasing (adjusted OR [aOR] 3.24; 95% CI 1.87–5.55) and High increasing (aOR 2.99; 95% CI 1.44–6.22) trajectories were independently associated with adverse neonatal outcomes. Addition of smaller twin FGV significantly improved discrimination beyond trajectory classification alone, and the combined model demonstrated moderate performance (development AUC 0.696; validation AUC 0.733). Exploratory machine learning analysis showed comparable performance (external validation AUC 0.710), and SHAP analysis identified smaller twin FGV as the most influential predictor.

**Conclusion:**

Integration of longitudinal discordance trajectories with fetal growth velocity may provide clinically relevant prognostic information beyond static measurements and may support individualized prenatal surveillance in twin pregnancies.

## Introduction

The global incidence of twin pregnancies has risen significantly over the past decades, now accounting for approximately 2–4% of all births, driven by the delay in childbearing age and the widespread application of assisted reproductive technologies (1). Twin gestations are associated with increased susceptibility to adverse perinatal outcomes, including preterm birth, low birth weight, and neonatal morbidity (2–4). Central to these risks is the challenge of unequal fetal growth, which often manifests as intertwin birthweight discordance or selective fetal growth restriction (sFGR) (5).

Currently, clinically significant intertwin growth discordance is conventionally defined using a single-point estimated fetal weight (EFW) difference of ≥20–25% (6, 7). However, the use of static, cross-sectional thresholds has notable limitations. First, it fails to capture the dynamic, longitudinal nature of fetal development, which may follow distinct phenotypic trajectories (8). Second, the standard singleton growth charts may lead to over-monitoring and over-diagnosis in twins, whose growth naturally slows in late gestation compared to singletons (9). Furthermore, existing longitudinal studies are often constrained by small sample sizes, a lack of external validation, or the failure to integrate discordance with growth velocity (10).

Fetal growth velocity (FGV), defined as the longitudinal rate of change in EFW over gestation, has emerged as a dynamic marker that may identify fetuses failing to achieve their intrinsic growth potential, even when absolute size remains within normal ranges (11). However, the clinical relevance of FGV is likely context-dependent, and its interplay with longitudinal patterns of intertwin discordance has not been systematically evaluated. Recent advances in Group-Based Trajectory Modeling (GBTM) offer a sophisticated unsupervised learning approach to identify latent growth phenotypes that traditional mean-based analyses might obscure (12).

There is a distinct clinical need to move beyond static measurements toward a multidimensional assessment of twin growth. This multicenter study aimed to characterize longitudinal intertwin discordance trajectories and FGV in a large cohort, evaluating their combined ability to refine prognostic assessment of adverse neonatal outcomes through both traditional logistic regression and interpretable machine learning frameworks.

## Materials and methods

### Study design and participants

This multicenter retrospective cohort study utilized data from two tertiary centers in Guangzhou, China: The First Affiliated Hospital of Sun Yat-Sen University (Center 1) and Guangdong Women and Children Hospital (Center 2). Both institutional ethics committees approved the study, and informed consent was waived due to the use of de-identified retrospective data.

Center 1 provided the development cohort (December 2013–December 2025), while Center 2 provided the external validation cohort (November 2021–March 2025). We included twin pregnancies (monochorionic and dichorionic) delivering at ≥28 weeks with ≥3 standardized ultrasound EFW measurements between 16 and 39 weeks (including at least one mid-trimester scan). Exclusion criteria were major congenital anomalies, chromosomal abnormalities, intrauterine fetal demise (IUFD), fetal reduction, miscarriage, incomplete clinical or ultrasound data. Pregnancies complicated by single or double IUFD were excluded because longitudinal assessment of intertwin growth discordance and fetal growth velocity could not be reliably performed after fetal death.

Ultrasound surveillance generally followed institutional protocols and contemporary twin pregnancy guidelines. Monochorionic twin pregnancies were typically monitored every 2 weeks from 16 weeks of gestation onward, whereas dichorionic twin pregnancies underwent ultrasound assessment approximately every 4 weeks after 20 weeks of gestation (13, 14). When abnormalities such as twin-to-twin transfusion syndrome (TTTS), twin anemia polycythemia sequence (TAPS), fetal growth restriction, or significant intertwin growth discordance were identified, surveillance frequency was intensified at the discretion of the managing specialists.

### Data collection and outcome definitions

Maternal, obstetric, and ultrasonographic variables were recorded, including maternal age, chorionicity, conception mode, gravidity, parity, pregnancy complications, and gestational age at each scan. Monochorionic-specific complications, such as TTTS, sFGR, and TAPS, were documented. A composite adverse neonatal outcome was defined to capture major clinically relevant neonatal morbidities associated with fetal growth impairment and prematurity, including neonatal respiratory distress syndrome (NRDS), mechanical ventilation, sepsis, necrotizing enterocolitis (NEC), or intraventricular hemorrhage (IVH) (15–17). Neonatal mortality, defined as death within the first 28 days after birth, was evaluated separately.

EFW was calculated using the Hadlock-3 formula, which has shown reliability in Chinese twin populations (9). Intertwin growth discordance was defined as: (EFW^larger^ − EFW^smaller^) / EFW^larger^ × 100%. FGV for each twin was defined as the slope of linear regression of EFW against gestational age (scaled to 10 g/week). Velocities for the larger and smaller twins were recorded as larger twin FGV (FGVA) and smaller twin FGV (FGVB), respectively, and the relative FGV difference (%) was calculated as: (FGVA − FGVB) / FGVA × 100%. Abnormal umbilical artery (UA) Doppler was defined as absent or reversed end-diastolic flow.

### Statistical analysis

Continuous variables were presented as median (interquartile range [IQR]) and categorical variables as counts (%). Differences between groups were assessed using Kruskal-Wallis tests for continuous variables, and chi-square or Fisher’s exact tests for categorical variables.

Longitudinal discordance trajectory modeling was performed using group-based trajectory modeling (GBTM) implemented under a maximum-likelihood framework, using repeated measurements of intertwin EFW discordance across gestational age. To avoid local optima, we employed a grid-search approach with 30 random starting values, allowing up to 100 iterations per fit. Model selection (2–5 classes) was based on Bayesian information criterion (BIC), entropy, posterior probability, minimum class size, and clinical interpretability. Final models were re-estimated with 500 iterations to ensure convergence. Bootstrap resampling (*n* = 500) assessed the stability of trajectory shapes.

Logistic regression examined associations between trajectory class, FGV metrics (FGVA, FGVB, and the relative FGV difference), last-visit discordance, and adverse neonatal outcomes (Center 1). Variable selection for prediction models was performed using Least Absolute Shrinkage and Selection Operator (LASSO) logistic regression in the development cohort. To evaluate whether the association between FGVB and adverse neonatal outcomes differed across discordance phenotypes, interaction effects between FGVB and trajectory class were assessed using Type 3 Wald chi-square tests. *P* for trend was calculated using numeric coding of trajectory categories.

To evaluate the incremental prognostic contribution of discordance trajectory class and FGVB, nested logistic regression models were compared in the development cohort. Model discrimination was assessed using area under the receiver operating characteristic curve (AUC), and differences in AUC were evaluated using DeLong’s test. Likelihood-ratio tests were additionally performed to compare nested models. The primary prediction model included trajectory class and FGVB, with Center 1 as the development cohort and Center 2 as the external validation cohort. Two extended models sequentially added abnormal UA Doppler and chorionicity.

To explore whether the prognostic value of FGVB differed across longitudinal discordance phenotypes, trajectory-stratified analyses were performed. Within each trajectory class, the discriminatory performance of FGVB for predicting composite adverse neonatal outcomes was evaluated using receiver operating characteristic (ROC) analysis, and AUC values with 95% confidence intervals (CIs) were estimated using DeLong’s method.

Separately, for exploratory machine learning analyses, Center 1 data were randomly divided into training (70%) and internal test (30%) subsets for XGBoost model development, followed by external validation using Center 2 data. Model interpretability was evaluated using SHapley Additive exPlanations (SHAP). XGBoost was selected because of its robust performance in structured clinical datasets and its ability to capture potential nonlinear relationships (18). It was considered exploratory because the primary objective of the study was clinical interpretation and hypothesis-driven modeling rather than optimization of predictive accuracy.

Sensitivity analyses included posterior probability-weighted regression, replication in the validation cohort, center-adjusted modeling, and inclusion of UA parameters (abnormal UA Doppler and intertwin UA pulsatility index [PI] discordance at last visit). Subgroup analyses stratified by chorionicity were performed in the development cohort to assess potential effect modification. Additional sensitivity analyses further adjusted for gestational age at delivery and hypertensive disorders of pregnancy (HDP) to evaluate whether associations between discordance trajectories, FGV metrics, and adverse neonatal outcomes remained independent of delivery timing and major pregnancy complications associated with adverse perinatal outcomes. To further assess the temporal robustness and minimize potential influence from the physiological slowing of twin fetal growth in late gestation, a restricted dataset including only ultrasound examinations up to 32 weeks was constructed while maintaining the requirement of at least three longitudinal measurements per fetus. Trajectory modeling and FGV estimation were then repeated using the same analytical framework as the primary analysis.

Analyses were conducted in R v4.3 using packages including lcmm, lme4, xgboost, and pROC. *P* < 0.05 was considered statistically significant.

## Results

### Identification of discordance trajectories

**Figure 1:**
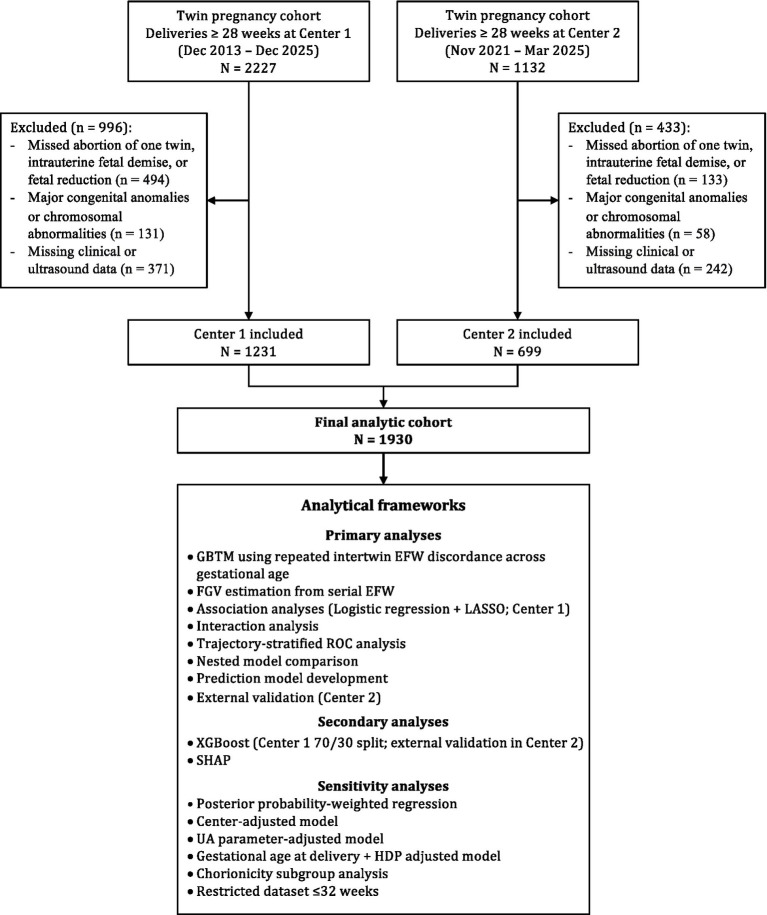
Study population flowchart and analytical framework. GBTM, group-based trajectory modeling; FGV, fetal growth velocity; EFW, estimated fetal weight; LASSO, Least Absolute Shrinkage and Selection Operator; ROC, receiver operating characteristic; SHAP, SHapley Additive exPlanations; UA, umbilical artery; HDP, hypertensive disorders of pregnancy.

**Figure 2:**
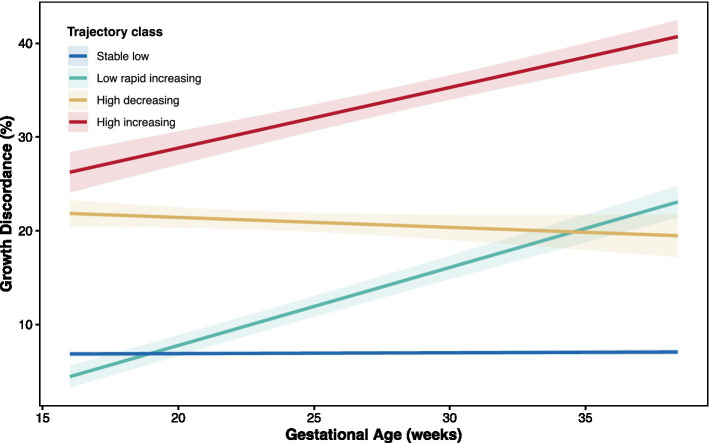
Discordance trajectories of intertwin growth identified by group-based trajectory modeling (GBTM). Solid lines represent model-predicted mean discordance for each trajectory class; shaded bands indicate 95% CIs.

A total of 1,930 twin pregnancies were included (Figure 1). Baseline maternal and obstetric characteristics showed modest differences between the development and validation cohorts (Table 1), reflecting variations in patient populations. Baseline characteristics according to adverse neonatal outcome status within each cohort are presented in Supplementary Table S1. Across both cohorts, pregnancies with adverse neonatal outcomes generally exhibited earlier delivery, lower birth weights, greater intertwin discordance, and less favorable fetal growth measures. GBTM identified four distinct longitudinal discordance trajectories (Figure 2; Supplementary Table S2): Stable low (*n* = 1,622, 84.1%), Low rapid increasing (*n* = 164, 8.5%), High decreasing (*n* = 99, 5.1%), and High increasing (*n* = 45, 2.3%). Bootstrap resampling demonstrated reproducibility of the major trajectory patterns, although variability in convergence and class proportions was observed across bootstrap samples, particularly for smaller trajectory groups (Supplementary Table S3). Spaghetti plots demonstrated clear separation of trajectory groups over gestation (Supplementary Figure S1).

**Table 1:** Baseline antenatal characteristics by center.

Variable	Overall (*N* = 1,930)	Center 1 (*N* = 1,231)	Center 2 (*N* = 699)	*p* value
Maternal characteristics				
Maternal age (years)	32.0 [29.0, 35.0]	32.0 [30.0, 36.0]	31.0 [28.0, 34.0]	<0.001
Gravidity	2 [1, 3]	2 [1, 2]	2 [1, 3]	0.221
Parity	0 [0, 1]	0 [0, 1]	0 [0, 1]	<0.001
Nulliparous	1,348 (69.8)	915 (74.3)	433 (61.9)	<0.001
Monochorionic twins	574 (29.7)	351 (28.5)	223 (31.9)	0.130
Assisted reproductive technology	1,072 (55.5)	758 (61.6)	314 (44.9)	<0.001
Prepregnancy BMI (kg/m^2^)	20.9 [19.3, 22.9]	20.8 [19.2, 22.8]	20.9 [19.4, 23.0]	0.227
Pregnancy complications				
Monochorionic twin complications	101 (5.2)	95 (7.7)	6 (0.9)	<0.001
Hyperglycemia	476 (24.7)	290 (23.6)	186 (26.6)	0.150
Hypertensive disorders of pregnancy	291 (15.1)	146 (11.9)	145 (20.7)	<0.001
Premature rupture of membranes	263 (13.6)	150 (12.2)	113 (16.2)	0.017
Ultrasound and fetal assessment				
Number of ultrasound examinations	6 [4, 7]	5 [4, 7]	6 [5, 8]	<0.001
Intertwin weight discordance at last visit (%)	7.7 [3.8, 13.7]	8.3 [4.3, 14.5]	6.9 [3.2, 12.2]	<0.001
Abnormal umbilical artery doppler	86 (4.5)	61 (5.0)	25 (3.6)	0.195
Fetal growth velocity—Larger twin (per 10 g/week)	13.1 [11.8, 14.6]	13.5 [12.2, 15.0]	12.4 [11.1, 13.7]	<0.001
Fetal growth velocity—Smaller twin (per 10 g/week)	11.8 [10.6, 13.2]	12.1 [10.9, 13.5]	11.4 [9.9, 12.5]	<0.001
Relative fetal growth velocity difference (%)	7.9 [4.0, 13.7]	8.3 [4.4, 14.5]	6.8 [3.4, 12.2]	<0.001

Continuous variables presented as median [IQR]; categorical as n (%). *p* values calculated using Kruskal-Wallis or χ^2^ tests.

### Fetal growth velocity across trajectories

**Figure 3:**
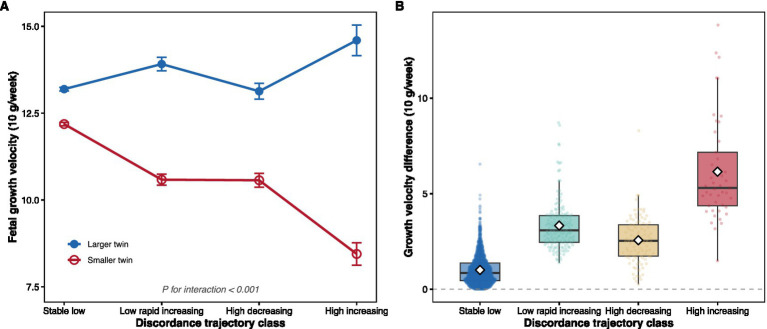
Fetal growth velocity (FGV) patterns across discordance trajectory classes. **(A)** Median growth velocity by twin type. Points represent median growth velocity with bootstrap 95% CIs (5,000 resamples) for larger and smaller twins across trajectory classes. *P* for interaction derived from linear mixed model with random intercept for twin pairs. *P* for interaction < 0.001. **(B)** Velocity difference (Larger twin FGV – smaller twin FGV) distribution. Boxplots with overlaid jitter points. Box = median and interquartile range; white diamond = mean; dashed line = zero difference. Due to heteroscedasticity across groups (Levene’s test *p* < 0.001), group comparisons were performed using robust F-test with HC3 standard errors, *p* < 0.001.

Median FGV for larger twins ranged from 127 to 144 g/week (Figure 3; Supplementary Table S4). In both the High decreasing and Low rapid increasing trajectories, growth was primarily driven by the reduced velocity of the smaller twin (Median 106–107 g/week) compared to the Stable low group (Median 121 g/week). The High increasing trajectory was characterized by the highest velocity in the larger twin (Median 144 g/week) and the lowest in the smaller twin (Median 83 g/week). Linear mixed-effects models with random intercepts for twin pairs showed that the difference in FGV between larger and smaller twins varied significantly across trajectory classes (*p* < 0.001 for interaction; Figure 3A). Velocity differences (FGVA − FGVB) were greatest in *High increasing* and *Low rapid increasing* classes (Figure 3B).

### Neonatal outcomes by trajectory class

**Figure 4:**
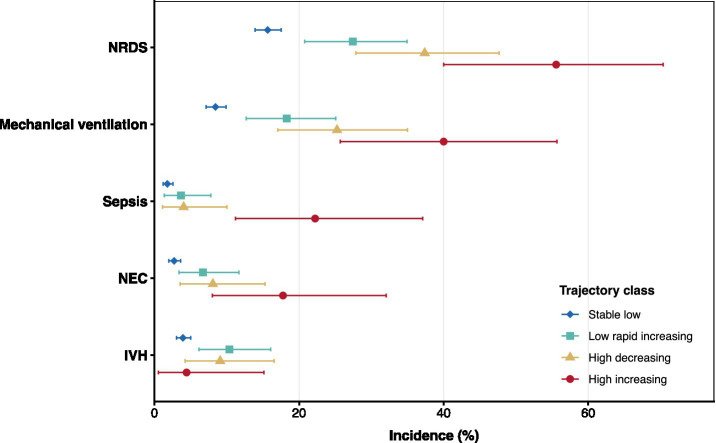
Adverse neonatal outcome incidence by discordance trajectory class. Points represent observed incidence rates and horizontal bars indicate exact binomial 95% CIs. P for trend values were calculated using Firth’s bias−reduced logistic regression to account for potential small−sample bias. NRDS: Neonatal respiratory distress syndrome; NEC: Necrotizing enterocolitis; IVH: Intraventricular hemorrhage. *P* for trend: NRDS <0.001, Mechanical ventilation <0.001, Sepsis <0.001, NEC < 0.001, IVH = 0.008.

Delivery outcomes varied significantly across trajectory classes (Table 2). The High increasing trajectory had the lowest median gestational age at delivery (34.0 weeks) and lowest birth weights (larger: 1,990 g; smaller: 1,340 g), alongside the highest intertwin birthweight discordance (33.3%). Rates of neonatal unit admission and composite adverse outcomes increased progressively from the Stable low to High increasing trajectory (Table 2; Figure 4). Trends for individual outcomes were generally consistent, except for IVH, which did not follow a monotonic pattern. Neonatal mortality occurred in 3 neonates, all of whom had already met criteria for the composite adverse neonatal outcomes. Center-stratified analyses demonstrated generally consistent increases in adverse neonatal outcomes across trajectory classes in both centers, although the magnitude of associations varied for some individual outcomes because of limited subgroup sample sizes (Supplementary Table S5).

**Table 2:** Delivery characteristics and neonatal outcomes by discordance trajectory class.

Variable	Overall (*N* = 1,930)	Stable low (*N* = 1,622)	Low rapid increasing (*N* = 164)	High decreasing (*N* = 99)	High increasing (*N* = 45)	*p* value
Delivery characteristics						
Cesarean delivery	1,848 (97.8)	1,552 (97.7)	156 (97.5)	96 (99.0)	44 (100.0)	0.621
Gestational age at delivery (weeks)	36.4 [35.4, 37.0]	36.6 [35.7, 37.1]	36.0 [34.4, 36.9]	35.4 [34.0, 36.4]	34.0 [32.0, 35.3]	<0.001
Birth weight—Larger twin (g)	2,510 [2,260, 2,720]	2,530 [2,300, 2,737]	2,500 [2,217, 2,740]	2,340 [2,060, 2,580]	1,990 [1,680, 2,380]	<0.001
Birth weight—Smaller twin (g)	2,255 [1,990, 2,480]	2,305 [2,080, 2,510]	1,985 [1,710, 2,242]	1,830 [1,560, 2,060]	1,340 [1,030, 1,680]	<0.001
Intertwin birth weight discordance (%)	8.6 [4.1, 15.6]	7.3 [3.4, 12.5]	20.0 [13.1, 26.6]	20.8 [16.0, 26.7]	33.3 [26.7, 38.1]	<0.001
Neonatal outcomes						
Admission to neonatal unit	1,286 (66.6)	1,014 (62.5)	136 (82.9)	92 (92.9)	44 (97.8)	<0.001
Composite adverse neonatal outcomes	473 (24.5)	336 (20.7)	62 (37.8)	49 (49.5)	26 (57.8)	<0.001

Continuous variables presented as median [IQR]; categorical as n (%). *p* values from χ^2^ tests. Composite adverse neonatal outcomes defined as neonatal respiratory distress syndrome (NRDS), mechanical ventilation, sepsis, necrotizing enterocolitis (NEC), or intraventricular hemorrhage (IVH).

### Logistic regression and interaction analyses

In the development cohort, LASSO regression (*λ* = λ_min) identified FGVB and discordance trajectory class as the strongest predictors of composite adverse neonatal outcomes. In univariable analyses, FGVA, relative FGV difference, and discordance at the last ultrasound examination were also significantly associated with adverse outcomes but were not retained in the final penalized model (Table 3). Compared with the Stable low group, the Low rapid increasing (Crude OR 2.46, 95% CI 1.55–3.82), High decreasing (Crude OR 4.42, 95% CI 2.61–7.42), and High increasing (Crude OR 6.81, 95% CI 3.55–13.22) trajectories were associated with increased risk. After adjusting for FGVB, the associations were attenuated, and the High decreasing (aOR 3.24, 95% CI 1.87–5.55) and High increasing (aOR 2.99, 95% CI 1.44–6.22) trajectories remained significant. Subgroup analyses stratified by chorionicity are presented in Supplementary Table S6. Although effect estimates varied across dichorionic and monochorionic pregnancies, formal interaction testing provided no evidence that chorionicity significantly modified the associations between discordance trajectory class, FGVB, and adverse neonatal outcomes (all *P* for interaction >0.05).

**Table 3:** Logistic regression models for composite adverse neonatal outcomes (development cohort).

Variables	Univariate analysis		Multivariable analysis	
	OR (95% CI)	*p* value	OR (95% CI)	*p* value
Discordance trajectory class (reference: Stable low)				
Low rapid increasing	2.46 (1.55–3.82)	<0.001	1.68 (1.03–2.68)	0.032
High decreasing	4.42 (2.61–7.42)	<0.001	3.24 (1.87–5.55)	<0.001
High increasing	6.81 (3.55–13.22)	<0.001	2.99 (1.44–6.22)	0.003
*P* for trend		<0.001		<0.001
Fetal growth velocity—Smaller twin (per 10 g/week)	0.74 (0.69–0.80)	<0.001	0.80 (0.74–0.87)	<0.001
Fetal growth velocity—Larger twin (per 10 g/week)	0.87 (0.81–0.93)	<0.001	-	-
Relative fetal growth velocity difference	1.04 (1.03–1.06)	<0.001	-	-
Discordance at last visit	1.05 (1.03–1.06)	<0.001	-	-

*P* for trend was calculated by modeling trajectory groups as an ordinal variable.

**Figure 5:**
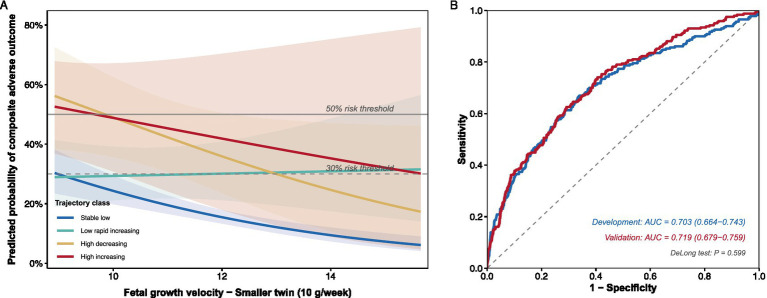
Integrated clinical risk stratification and model discrimination. **(A)** Marginal effects plot showing the interaction between discordance trajectory class and fetal growth velocity of the smaller twin (FGVB) on the predicted probability of composite adverse neonatal outcome in the development cohort. Solid lines represent predicted probabilities with 95% CIs (shaded bands) from a logistic regression model including the trajectory class × FGVB interaction term. Horizontal dashed and solid lines indicate 30 and 50% risk thresholds, respectively. **(B)** ROC curves for the extended logistic regression model incorporating trajectory class, FGVB, abnormal umbilical artery Doppler and chorionicity, in the development (blue) and validation (red) cohorts.

The overall interaction between FGVB and trajectory class did not reach statistical significance in the development cohort (*p* = 0.059) nor in the validation cohort (*p* = 0.742; Supplementary Table S7); nevertheless, the specific interaction term for FGVB × Low rapid increasing trajectory was significant in the development cohort (OR 1.35, 95% CI 1.08–1.69, *p* = 0.008; Supplementary Table S7; Figure 5A; Supplementary Figure S2), promoting exploratory trajectory-stratified analyses to evaluate the discriminatory performance of FGVB within each trajectory class. FGVB retained moderate discriminatory ability within the High decreasing (AUC 0.667, 95% CI 0.524–0.810) and Stable low trajectories (AUC 0.653, 95% CI 0.601–0.705), whereas its performance was limited within the Low rapid increasing trajectory (AUC 0.482, 95% CI 0.355–0.609; Supplementary Table S8).

### Predictive modeling: logistic regression and machine learning

To evaluate the incremental predictive values of trajectory class and FGVB, nested logistic regression models were compared (Supplementary Table S9). Addition of FGVB to trajectory class significantly improved model discrimination (AUC 0.616 vs. 0.696; ΔAUC = 0.080; *p* < 0.001), whereas addition of trajectory class to FGVB alone produced a smaller but statistically significant improvement (AUC 0.676 vs. 0.696; ΔAUC = 0.020; *p* = 0.045). The primary prediction model therefore included both trajectory class and FGVB and demonstrated moderate discrimination (development AUC 0.696; validation AUC 0.733; Supplementary Table S10). Addition of abnormal UA Doppler and chorionicity yielded minimal gains in the development cohort but did not consistently improve discrimination in external validation (development AUC 0.703; validation AUC 0.719; Figure 5B). The exploratory XGBoost model achieved similar predictive performance (internal test AUC 0.738; external validation AUC 0.710; Supplementary Table S11). Feature importance analyses identified FGVB as the most influential predictor and suggested a non-linear inverse relationship between FGVB and predicted adverse outcome risk, with lower FGVB values associated with progressively higher model-predicted risk (Supplementary Figure S3).

### Sensitivity analyses

Sensitivity analyses, including posterior probability-weighted regression, validation cohort replication, center-adjusted logistic models, and incorporation of UA parameters (abnormal UA Doppler and intertwin UA PI discordance at last visit), yielded findings broadly consistent with the primary analyses (Supplementary Tables S12, S13). Additional adjustment for gestational age at delivery and HDP attenuated associations for the Low rapid increasing and High increasing trajectories as well as FGVB, whereas the High decreasing trajectory remained independently associated with adverse neonatal outcomes (aOR 2.45, 95% CI 1.32–4.47). Gestational age at delivery remained strongly associated with adverse neonatal outcomes (Supplementary Table S14). To further evaluate temporal robustness, analyses were restricted to ultrasound data ≤32 weeks. Four trajectory classes remained identifiable with stable classification performance, and discordance trajectories continued to show associations with adverse outcomes. In multivariable models, Low rapid increasing (aOR 2.86, 95% CI 1.65–4.87), High decreasing (aOR 4.49, 95% CI 2.47–8.14), and High increasing (aOR 4.95, 95% CI 2.57–9.57) trajectories remained significantly associated with increased risk, whereas the effect of FGVB was attenuated and no longer statistically significant (aOR 0.95, 95% CI 0.87–1.04; Supplementary Table S15).

## Discussion

This multicenter study identified four distinct longitudinal intertwin discordance trajectories—Stable low, Low rapid increasing, High decreasing, and High increasing—which, when considered alongside smaller twin FGV, were significantly associated with adverse neonatal outcomes. These findings suggest that longitudinal assessment of discordance may provide additional prognostic information beyond conventional static discordance thresholds.

While static thresholds (e.g., discordance ≥20–25%, or early progressive discordance defined by fixed 10% cutoffs) remain commonly referenced in clinical practice, such approaches may not adequately capture the temporal evolution of fetal growth divergence (10, 19). Our identification of four trajectory phenotypes provides a more nuanced characterization of discordance progression and is broadly consistent with prior trajectory-based investigations. Prasad et al. applied an unsupervised learning algorithm in a US-based cohort of 823 twin pregnancies and described five discordance trajectories, reporting increased neonatal morbidity among pregnancies with persistently elevated discordance (15). Similarly, in our cohort, pregnancies assigned to the High increasing and High decreasing trajectories experienced substantially higher rates of adverse outcomes than those in the Stable low trajectory. Sensitivity analyses incorporating gestational age at delivery and HDP attenuated several of the observed associations, suggesting that delivery timing and pregnancy complications may partly contribute to the relationship between fetal growth patterns and neonatal outcomes. Nevertheless, the association for the High decreasing trajectory persisted after adjustment, indicating that this phenotype may capture risk information beyond these factors. One possible explanation is that the consequences of early fetal exposure to placental dysfunction or unequal placental sharing may not be fully reversed even when intertwin growth discordance later decreases (20–22). Sensitivity analyses, including restriction to ultrasound examinations ≤32 weeks, yielded generally consistent trajectory–outcome associations, suggesting that discordance phenotypes may be identifiable earlier in gestation (21, 23).

Notably, nested model comparisons demonstrated that FGVB provided incremental prognostic information beyond trajectory classification alone, whereas trajectory class also provided a statistically significant, although quantitatively modest improvement beyond FGVB alone. Prior studies have suggested that growth velocity may identify fetuses failing to achieve their intrinsic growth potential even when absolute fetal size remains within normal ranges, and may therefore capture aspects of placental dysfunction not fully reflected by cross-sectional biometric measurements (6, 11, 24, 25). However, the attenuation of its association after adjustment for gestational age at delivery and HDP suggests that part of its prognostic value may operate through pathways related to pregnancy complications and delivery timing. Exploratory interaction analyses suggested that the association between smaller twin growth velocity and adverse neonatal outcomes may vary across trajectory patterns in the development cohort; however, this finding was not replicated in the external validation cohort and should therefore be regarded as hypothesis-generating. Together, these findings suggest that longitudinal discordance trajectories and smaller twin growth velocity may capture related but non-identical dimensions of fetal growth physiology.

Trajectory-stratified ROC analyses further suggested that the discriminatory performance of FGVB differed across discordance phenotypes. FGVB demonstrated moderate discrimination within the Stable low and High decreasing trajectories but showed limited performance within the Low rapid increasing trajectory. Although these subgroup analyses were exploratory and some trajectory groups were relatively small, the findings may indicate heterogeneity in predictive performance of growth velocity across discordance phenotypes. Conventional logistic regression models demonstrated moderate and reproducible discrimination across development and validation cohorts, indicating that the combination of discordance trajectory and smaller twin FGV captured relevant prognostic information using a clinically interpretable framework. Exploratory machine learning analysis yielded similar predictive performance, suggesting that the principal predictive signal may already be adequately represented by the trajectory-based regression model. Although XGBoost was included to explore potential nonlinear relationships, it did not provide meaningful incremental predictive benefit over traditional regression-based modeling.

From a clinical perspective, these findings suggest that identification of high-risk trajectory patterns, particularly the High increasing and High decreasing groups, may help identify pregnancies warranting closer surveillance and earlier consideration of clinical intervention when otherwise indicated (5, 14, 26), whereas persistently low-risk patterns may support more conservative follow-up and potentially reduce unnecessary intervention. Prospective validation will be required before clinical implementation.

The strengths of this study include its multicenter design, trajectory model validation procedures, independent external validation cohort, and complementary use of interpretable machine learning methods. Several limitations should also be acknowledged. First, the retrospective study design introduces the possibility of residual confounding and limits causal inference. Second, ultrasound surveillance intervals varied across pregnancies according to clinical indications, which may have influenced estimation of fetal growth velocity and trajectory assignment. Third, pregnancies complicated by IUFD or fetal reduction were excluded because longitudinal assessment of discordance trajectories and fetal growth velocity could not be reliably continued thereafter. Consequently, the findings primarily apply to ongoing twin pregnancies undergoing serial ultrasound surveillance and may not be generalizable to pregnancies complicated by fetal death. Finally, some variability in bootstrap convergence and class proportions was observed among smaller trajectory groups, reflecting the limited sample size available for certain latent classes and the inherent uncertainty of latent class modeling.

## Conclusion

Longitudinal intertwin discordance trajectories, particularly when evaluated alongside smaller twin fetal growth velocity, were associated with adverse neonatal outcomes in twin pregnancies. These dynamic ultrasound metrics may provide additional prognostic information beyond static measures and may guide more individualized surveillance strategies.

## Data Availability

The original contributions presented in the study are included in the article/Supplementary material, further inquiries can be directed to the corresponding authors.

## Glossary

aOR: adjusted OR
AUC: Area under the receiver operating characteristic curve
BIC: Bayesian information criterion
CI: confidence interval
EFW: estimated fetal weight
FGV: fetal growth velocity
FGVA: fetal growth velocity of the larger twin
FGVB: fetal growth velocity of the smaller twin
GBTM: Group-based trajectory modeling
HDP: hypertensive disorders of pregnancy
IQR: interquartile range
IUFD: intrauterine fetal demise
IVH: intraventricular hemorrhage
LASSO: Least Absolute Shrinkage and Selection Operator
NEC: necrotizing enterocolitis
NRDS: neonatal respiratory distress syndrome
PI: pulsatility index
ROC: receiver operating characteristic
sFGR: selective fetal growth restriction
SHAP: SHapley Additive exPlanations
TAPS: twin anemia polycythemia sequence
TTTS: twin-to-twin transfusion syndrome
UA: umbilical artery
