# Supplementary material for: Twin growth discordance trajectories and fetal growth velocity for predicting adverse neonatal outcomes: a multicenter cohort study
Source: Front Med (Lausanne). 2026 Jun 17;13:1851509. doi: 10.3389/fmed.2026.1851509 (PMC13319096; doi:10.3389/fmed.2026.1851509)

Supplementary Materials

**Twin Growth Discordance Trajectories and Fetal Growth Velocity for Predicting Adverse Neonatal Outcomes: A Multicenter Cohort Study**

**Authors:** Minhuan Lin^1, 2†^, Xuewen Huang^3†^, Yuheng Zhou^4^, Shuting Xia^1, 2^, Dongmei Duan^4^, Yingnan Ye^1, 2^, Yiqing Chen^1, 2^, Linhuan Huang^1, 2*^, Jiying Wen^4*^, Yanmin Luo^1, 2*^

^†^ These authors contributed equally to this work and share first authorship.

**Author Affiliations:**

^1^ Department of Obstetrics & Gynecology, The First Affiliated Hospital of Sun Yat-Sen University, Guangzhou, China

^2^ ﻿Guangdong Provincial Clinical Research Center for Obstetrical and Gynecological Diseases, Guangzhou, China

^3^﻿ Department of Obstetrics & Gynecology, Zhongshan City People's Hospital, Zhongshan, China

^4^﻿ Guangdong Women and Children Hospital, Guangzhou, China

**Correspondence:** Yanmin Luo, luoyanm@mail.sysu.edu.cn; Jiying Wen, wenhu77@163.com; Linhuan Huang, hlhuan@mail.sysu.edu.cn

## Table S1. Baseline characteristics according to adverse neonatal outcome status in the development and validation cohorts

| **Variable** | **Center 1**  **(N = 1231)** | | | **Center 2**  **(N = 699)** | | |
| --- | --- | --- | --- | --- | --- | --- |
|  | **No adverse outcome**  **(N = 1001)** | **Adverse outcome**  **(N = 230)** | ***P* Value** | **No adverse outcome**  **(N = 456)** | **Adverse outcome**  **(N = 243)** | ***P* Value** |
| **Maternal characteristics** |  |  |  |  |  |  |
| Maternal age (years) | 33.0 [30.0, 36.0] | 32.0 [29.0, 35.0] | 0.256 | 31.0 [28.0, 34.0] | 31.0 [28.0, 35.0] | 0.262 |
| Gravidity | 2 [1, 2] | 2 [1, 2] | 0.482 | 2 [1, 3] | 2 [1, 3] | 0.581 |
| Parity | 0 [0, 1] | 0 [0, 0] | 0.801 | 0 [0, 1] | 0 [0, 1] | 0.537 |
| Nulliparous | 743 (74.2) | 172 (74.8) | 0.928 | 278 (61.0) | 155 (63.8) | 0.516 |
| Monochorionic twins | 244 (24.4) | 107 (46.5) | <0.001 | 124 (27.2) | 99 (40.7) | <0.001 |
| Assisted reproductive technology | 640 (63.9) | 118 (51.3) | 0.001 | 202 (44.3) | 112 (46.1) | 0.709 |
| Prepregnancy BMI (kg/m²) | 20.8 [19.3, 22.8] | 20.8 [19.1, 23.3] | 0.863 | 21.0 [19.2, 23.1] | 20.9 [19.5, 23.0] | 0.999 |
| **Pregnancy complications** |  |  |  |  |  |  |
| Monochorionic twin complications | 46 (4.6) | 49 (21.3) | <0.001 | 4 (0.9) | 2 (0.8) | 1.000 |
| Hyperglycemia | 217 (21.7) | 73 (31.7) | 0.002 | 110 (24.1) | 76 (31.3) | 0.051 |
| Hypertensive disorders of pregnancy | 100 (10.0) | 46 (20.0) | <0.001 | 84 (18.4) | 61 (25.1) | 0.048 |
| Premature rupture of membranes | 103 (10.3) | 47 (20.4) | <0.001 | 46 (10.1) | 67 (27.6) | <0.001 |
| **Ultrasound and fetal assessment** |  |  |  |  |  |  |
| Number of ultrasound examinations | 5 [4, 7] | 6 [4, 9] | <0.001 | 6 [5, 7] | 6 [4, 8] | 0.154 |
| Intertwin weight discordance at last visit (%) | 7.8 [4.1, 13.7] | 10.2 [5.3, 19.6] | <0.001 | 6.1 [2.9, 11.0] | 8.4 [3.9, 16.1] | <0.001 |
| Abnormal umbilical artery doppler | 28 (2.8) | 33 (14.3) | <0.001 | 9 (2.0) | 16 (6.6) | 0.004 |
| Fetal growth velocity - Larger twin (per 10 g/week) | 13.7 [12.4, 15.1] | 12.7 [11.5, 14.5] | <0.001 | 12.8 [11.7, 13.9] | 11.5 [10.0, 12.9] | <0.001 |
| Fetal growth velocity - Smaller twin (per 10 g/week) | 12.3 [11.1, 13.6] | 11.0 [9.4, 12.4] | <0.001 | 11.9 [10.8, 12.8] | 10.3 [8.9, 11.8] | <0.001 |
| Relative fetal growth velocity difference (%) | 8.0 [4.2, 13.4] | 10.0 [5.7, 20.8] | <0.001 | 5.9 [3.3, 10.3] | 8.5 [4.0, 15.8] | <0.001 |
| **Delivery characteristics** |  |  |  |  |  |  |
| Cesarean delivery | 960 (99.1) | 219 (98.6) | 0.845 | 443 (97.1) | 226 (93.0) | 0.017 |
| Gestational age at delivery (weeks) | 36.7 [36.0, 37.0] | 34.7 [33.6, 35.9] | <0.001 | 37.0 [36.3, 37.3] | 34.6 [33.1, 35.9] | <0.001 |
| Birth weight - Larger twin (g) | 2560 [2360, 2740] | 2200 [1970, 2477] | <0.001 | 2640 [2440, 2840] | 2240 [1900, 2490] | <0.001 |
| Birth weight - Smaller twin (g) | 2320 [2100, 2510] | 1920 [1612, 2170] | <0.001 | 2380 [2160, 2602] | 1930 [1680, 2200] | <0.001 |
| Intertwin birth weight discordance (%) | 7.9 [3.7, 14.5] | 12.3 [5.6, 20.5] | <0.001 | 8.3 [3.6, 14.2] | 10.6 [5.7, 17.8] | 0.001 |
| **Trajectory class (%)** |  |  |  |  |  |  |
| Stable low | 870 (86.9) | 149 (64.8) | <0.001 | 416 (91.2) | 187 (77.0) | <0.001 |
| Low rapid increasing | 76 (7.6) | 32 (13.9) |  | 26 (5.7) | 30 (12.3) |  |
| High decreasing | 37 (3.7) | 28 (12.2) |  | 13 (2.9) | 21 (8.6) |  |
| High increasing | 18 (1.8) | 21 (9.1) |  | 1 (0.2) | 5 (2.1) |  |
| Data are median [IQR] for continuous variables; n (%) for categorical variables.  *P* values were calculated within each cohort comparing adverse outcome vs. no adverse outcome using Wilcoxon rank-sum test (continuous) and χ² or Fisher's exact test (categorical).  Adverse neonatal outcome defined as composite adverse neonatal outcomes, including **neonatal respiratory distress syndrome (NRDS)**, mechanical ventilation, sepsis, n**ecrotizing enterocolitis (NEC), and intraventricular hemorrhage (IVH).** | | | | | | |

## Table S2. Trajectory Model Comparison

| **Classes** | **BIC** | **Min class (%)** | **Entropy** | **APP** | **Clinical interpretability** |
| --- | --- | --- | --- | --- | --- |
| 1 | 75,828.55 | - | - | - | Reference |
| 2 | 74,874.56 | 7.3 | 0.952 | 0.986 | Limited, only 2 groups identified |
| 3 | 74,466.17 | 4.9 | 0.892 | 0.952 | Good, clinically meaningful 3-group solution |
| **4** | **74,205.53** | **2.3** | **0.913** | **0.953** | **Best, captures unique high-decreasing pattern** |
| 5 | 74,053.57 | 1.0 | 0.914 | 0.948 | Overfitted, small class (n=19, 1.0%) |
| Class proportions: 2-class: 7.3% / 92.7%; 3-class: 4.9% / 8.8% / 86.3%; 4-class: 2.3% / 5.1% / 8.5% / 84.1%; 5-class: 1.0% / 3.6% / 4.1% / 9.1% / 82.2%. Abbreviations: BIC, Bayesian Information Criterion; Min class (%), minimum class proportion; APP, average posterior probability. | | | | | |

## Table S3. Trajectory Stability via Bootstrap Resampling

| **Trajectory Class** | **Original** | | **Bootstrap** | |
| --- | --- | --- | --- | --- |
|  | **Slope** | **%** | **Mean Slope ± SD** | **Mean % ± SD** |
| Stable low | 0.009 | 84.0 | 0.036 ± 0.055 | 59.6 ± 36.2 |
| Low rapid increasing | 0.832 | 8.5 | 0.850 ± 0.120 | 7.2 ± 2.9 |
| High decreasing | -0.106 | 5.1 | -0.138 ± 0.147 | 29.3 ± 36.7 |
| High increasing | 0.646 | 2.3 | 0.676 ± 0.099 | 3.9 ± 2.6 |
| Slope units: change in discordance (%) per week of gestation. Bootstrap estimates presented as mean ± standard deviation based on 500 resamples with replacement at the individual level. The model converged in 326 out of 500 bootstrap samples (65.2%). Non-convergence in a subset of resamples is expected in latent class models due to the complex likelihood surface and does not necessarily indicate model instability. | | | | |

## Table S4. Baseline Characteristics by Discordance Trajectory Class

| **Variable** | **Overall (N = 1930)** | **Stable low (N = 1622)** | **Low rapid increasing (N = 164)** | **High decreasing (N = 99)** | **High increasing (N = 45)** | ***P* Value** |
| --- | --- | --- | --- | --- | --- | --- |
| **Maternal characteristics** |  |  |  |  |  |  |
| Maternal age (years) | 32.0 [29.0, 35.0] | 32.0 [29.0, 35.0] | 31.0 [29.0, 35.2] | 31.0 [27.5, 34.0] | 30.0 [28.0, 36.0] | 0.012 |
| Gravidity | 2 [1, 3] | 2 [1, 3] | 2 [1, 2] | 1 [1, 2] | 2 [1, 3] | 0.811 |
| Parity | 0 [0, 1] | 0 [0, 1] | 0 [0, 1] | 0 [0, 1] | 0 [0, 1] | 0.859 |
| Nulliparous | 1348 (69.8) | 1131 (69.7) | 118 (72.0) | 68 (68.7) | 31 (68.9) | 0.932 |
| Monochorionic twins | 574 (29.7) | 427 (26.3) | 46 (28.0) | 63 (63.6) | 38 (84.4) | <0.001 |
| Assisted reproductive technology | 1072 (55.5) | 937 (57.8) | 92 (56.1) | 32 (32.3) | 11 (24.4) | <0.001 |
| Prepregnancy BMI (kg/m²) | 20.9 [19.3, 22.9] | 21.0 [19.4, 22.9] | 20.9 [19.3, 23.1] | 20.1 [18.7, 22.9] | 20.7 [19.1, 22.6] | 0.069 |
| **Pregnancy complications** |  |  |  |  |  |  |
| Hyperglycemia | 476 (24.7) | 399 (24.6) | 32 (19.5) | 32 (32.3) | 13 (28.9) | 0.116 |
| Hypertensive disorders of pregnancy | 291 (15.1) | 235 (14.5) | 29 (17.7) | 16 (16.2) | 11 (24.4) | 0.214 |
| Premature rupture of membranes | 263 (13.6) | 225 (13.9) | 25 (15.2) | 11 (11.1) | 2 (4.4) | 0.240 |
| Monochorionic twin complications | 101 (5.2) | 25 (1.5) | 19 (11.6) | 30 (30.3) | 27 (60.0) | <0.001 |
| **Ultrasound and fetal assessment** |  |  |  |  |  |  |
| Number of ultrasound examinations | 6 [4, 7] | 5 [4, 7] | 6 [4, 8] | 7 [5, 10] | 7 [6, 10] | <0.001 |
| Intertwin weight discordance at last visit (%) | 7.7 [3.8, 13.7] | 6.4 [3.2, 10.6] | 21.4 [17.7, 24.6] | 19.4 [14.9, 25.1] | 35.4 [31.0, 42.6] | <0.001 |
| Abnormal UA doppler | 86 (4.5) | 26 (1.6) | 10 (6.1) | 23 (23.2) | 27 (60.0) | <0.001 |
| Fetal growth velocity - Larger twin (per 10 g/week) | 13.1 [11.8, 14.6] | 13.0 [11.7, 14.5] | 13.9 [12.1, 15.3] | 12.7 [11.7, 14.3] | 14.4 [11.9, 16.4] | <0.001 |
| Fetal growth velocity - Smaller twin (per 10 g/week) | 11.8 [10.6, 13.2] | 12.1 [10.9, 13.3] | 10.7 [9.3, 11.8] | 10.6 [9.3, 11.6] | 8.3 [7.3, 9.3] | <0.001 |
| Fetal growth velocity difference (%) | 7.9 [4.0, 13.7] | 6.5 [3.5, 10.4] | 22.1 [18.6, 26.0] | 18.9 [14.0, 25.0] | 35.8 [32.4, 46.2] | <0.001 |
| Data are presented as median [IQR] or n (%). Between-group comparisons: continuous variables were compared using Kruskal-Wallis tests; categorical variables were compared using χ² tests or Fisher's exact tests. | | | | | | |

## Table S5. Neonatal Outcomes by Discordance Trajectory Class and Center

| **Outcome** | **Stable low (N = 1622)** | **Low rapid increasing (N = 164)** | **High decreasing (N = 99)** | **High increasing (N = 45)** | ***P* value** |
| --- | --- | --- | --- | --- | --- |
| **Neonatal respiratory distress syndrome (NRDS)** | **254 (15.7%)** | **45 (27.4%)** | **37 (37.4%)** | **25 (55.6%)** | **<0.001** |
| Center 1 | 105 (10.3%) | 24 (22.2%) | 22 (33.8%) | 20 (51.3%) | <0.001 |
| Center 2 | 149 (24.7%) | 21 (37.5%) | 15 (44.1%) | 5 (83.3%) | <0.001 |
| **Mechanical ventilation** | **137 (8.4%)** | **30 (18.3%)** | **25 (25.3%)** | **18 (40.0%)** | **<0.001** |
| Center 1 | 96 (9.4%) | 24 (22.2%) | 19 (29.2%) | 17 (43.6%) | <0.001 |
| Center 2 | 41 (6.8%) | 6 (10.7%) | 6 (17.6%) | 1 (16.7%) | 0.053 |
| **Sepsis** | **29 (1.8%)** | **6 (3.7%)** | **4 (4.0%)** | **10 (22.2%)** | **<0.001** |
| Center 1 | 13 (1.3%) | 4 (3.7%) | 2 (3.1%) | 8 (20.5%) | <0.001 |
| Center 2 | 16 (2.7%) | 2 (3.6%) | 2 (5.9%) | 2 (33.3%) | 0.010 |
| **Necrotizing enterocolitis (NEC)** | **44 (2.7%)** | **11 (6.7%)** | **8 (8.1%)** | **8 (17.8%)** | **<0.001** |
| Center 1 | 27 (2.6%) | 9 (8.3%) | 6 (9.2%) | 8 (20.5%) | <0.001 |
| Center 2 | 17 (2.8%) | 2 (3.6%) | 2 (5.9%) | 0 (0.0%) | 0.432 |
| **Intraventricular hemorrhage (IVH)** | **64 (3.9%)** | **17 (10.4%)** | **9 (9.1%)** | **2 (4.4%)** | **<0.001** |
| Center 1 | 8 (0.8%) | 0 (0.0%) | 1 (1.5%) | 1 (2.6%) | 0.258 |
| Center 2 | 56 (9.3%) | 17 (30.4%) | 8 (23.5%) | 1 (16.7%) | <0.001 |
| Data are presented as n (%). *P* values from χ² or Fisher exact tests. | | | | | |

| **Table S6. Subgroup Analysis by Chorionicity (Development Cohort)** | | | | | | |
| --- | --- | --- | --- | --- | --- | --- |
| **Variable** | **Stratification level** | **Total N** | **Events, n (%)** | **OR (95% CI)** | ***P* value** | ***P* for interaction** |
| Discordance trajectory class (reference: Stable low) | |  |  |  |  |  |
| Low rapid increasing | Overall | 1,231 | 230 (18.7%) | 1.68 (1.03–2.68) | 0.032 | 0.564 |
|  | Dichorionic | 880 | 123 (14.0%) | 2.23 (1.25–3.90) | 0.005 |  |
|  | Monochorionic | 351 | 107 (30.5%) | 1.13 (0.44–2.73) | 0.784 |  |
| High decreasing | Overall | 1,231 | 230 (18.7%) | 3.24 (1.87–5.55) | <0.001 |  |
|  | Dichorionic | 880 | 123 (14.0%) | 2.76 (1.07–6.53) | 0.026 |  |
|  | Monochorionic | 351 | 107 (30.5%) | 2.68 (1.31–5.47) | 0.006 |  |
| High increasing | Overall | 1,231 | 230 (18.7%) | 2.99 (1.44–6.22) | 0.003 |  |
|  | Dichorionic | 880 | 123 (14.0%) | 1.03 (0.05–7.54) | 0.978 |  |
|  | Monochorionic | 351 | 107 (30.5%) | 2.06 (0.87–4.92) | 0.100 |  |
| Fetal growth velocity - Smaller twin (per 10 g/week) | Overall | 1,231 | 230 (18.7%) | 0.80 (0.74–0.87) | <0.001 | 0.184 |
|  | Dichorionic | 880 | 123 (14.0%) | 0.86 (0.77–0.95) | 0.005 |  |
|  | Monochorionic | 351 | 107 (30.5%) | 0.76 (0.66–0.87) | <0.001 |  |
| Multivariable logistic regression models were adjusted for trajectory class and fetal growth velocity of the smaller twin.  *P* for interaction was derived from likelihood ratio tests comparing models with and without the interaction term (chorionicity × predictor). | | | | | | |

## Table S7. Logistic Regression Models with FGVB × Trajectory Interaction

| **Variables** | **Development Cohort (Center 1)** | | **Validation Cohort (Center 2)** | | |
| --- | --- | --- | --- | --- | --- |
|  | **OR (95% CI)** | ***P* value** | **OR (95% CI)** | ***P* value** | |
| Fetal growth velocity - Smaller twin (per 10 g/week) | 0.75 (0.68–0.83) | <0.001 | 0.62 (0.55–0.69) | <0.001 | |
| Discordance trajectory class (reference: Stable low) |  |  |  |  | |
| Low rapid increasing | 0.06 (0.01–0.75) | 0.030 | 1.23 (0.03–87.01) | 0.917 | |
| High decreasing | 2.64 (0.12–77.91) | 0.555 | 10.40 (0.03–20291.98) | 0.496 | |
| High increasing | 0.72 (0.04–15.50) | 0.826 | 0.00 (0.00–15873.90) | 0.414 | |
| Interaction terms (FGVB × Trajectory) |  |  |  |  | |
| FGVB × Low rapid increasing | 1.35 (1.08–1.69) | 0.008 | 1.03 (0.69–1.45) | 0.885 | |
| FGVB × High decreasing | 1.01 (0.74–1.34) | 0.933 | 0.85 (0.41–1.52) | 0.622 | |
| FGVB × High increasing | 1.15 (0.82–1.56) | 0.374 | 2.70 (0.36–100.61) | 0.381 | |
| Overall *P* for interaction |  | 0.059 |  | 0.742 | |
| Models include main effects for discordance trajectory class and FGVB, plus their interaction term.  Overall interaction *P* values were calculated using Type 3 Wald chi-square tests. | | | | |  |

## Table S8. Trajectory-stratified Discriminatory Performance of FGVB

| Trajectory class | AUC of FGVB | DeLong *P* |
| --- | --- | --- |
| Stable low | 0.653 (0.601–0.705) | <0.001 |
| Low rapid increasing | 0.482 (0.355–0.609) | 0.781 |
| High decreasing | 0.667 (0.524–0.810) | 0.022 |
| High increasing | 0.606 (0.423–0.788) | 0.255 |
| Within each trajectory class, the FGVB model includes fetal growth velocity of the smaller twin as a continuous predictor.  AUC values are presented with 95% confidence intervals using DeLong's method.  DeLong *P* compares the ROC curve of the FGVB model against the null (intercept-only) model.  Abbreviations: AUC, area under the curve; FGVB, fetal growth velocity of the smaller twin (per 10 g/week). | | |

| **Table S9. Nested Model Comparison in the Development Cohort** | | | | |
| --- | --- | --- | --- | --- |
| Comparison | Reduced model AUC | Full model AUC | ΔAUC | LRT *P* |
| Trajectory only → Trajectory + FGVB | 0.616 (0.582–0.649) | 0.696 (0.656–0.736) | +0.0806 (P <0.001) | <0.001 |
| FGVB only → FGVB + trajectory | 0.676 (0.634–0.718) | 0.696 (0.656–0.736) | +0.0201 (P = 0.045) | <0.001 |
| Reduced model AUC is from Model 1 (trajectory only) or Model 2 (FGVB only).  Full model AUC is from Model 3 (trajectory + FGVB).  ΔAUC = Full model AUC − Reduced model AUC; *P* values for ΔAUC are from DeLong's test.  LRT *P* values are from likelihood ratio tests comparing reduced vs full model.  Abbreviations: FGVB, fetal growth velocity of the smaller twin (per 10 g/week). | | | | |

## Table S10. Model Performance for Logistic Regression Models in Development and Validation Cohorts

| **Dataset** | **Model** | **AUC (95% CI)** | **Sens (%)** | **Spec (%)** | **PPV (%)** | **NPV (%)** | **Brier** |
| --- | --- | --- | --- | --- | --- | --- | --- |
| Development | Base Model | 0.696 (0.656–0.736) | 59.1 | 74.1 | 34.4 | 88.8 | 0.138 |
| Development | Extended Model 1 | 0.696 (0.655–0.736) | 59.1 | 73.7 | 34.1 | 88.7 | 0.137 |
| Development | Extended Model 2 | 0.703 (0.664–0.743) | 67.0 | 66.1 | 31.2 | 89.7 | 0.136 |
| Validation | Base Model | 0.733 (0.694–0.773) | 66.7 | 68.2 | 52.8 | 79.3 | 0.220 |
| Validation | Extended Model 1 | 0.734 (0.694–0.773) | 67.1 | 67.5 | 52.4 | 79.4 | 0.221 |
| Validation | Extended Model 2 | 0.719 (0.679–0.759) | 73.3 | 60.1 | 49.4 | 80.8 | 0.220 |
| Base Model = Trajectory class + FGVB; Extended Model 1 = Base Model + Abnormal UA Doppler; Extended Model 2 = Extended Model 1 + Chorionicity.  All models were fitted using the development dataset (Center 1). Validation metrics were calculated using the same models applied to the validation dataset (Center 2). Optimal thresholds were derived using Youden index from the development dataset for each model.  Abbreviations: AUC, area under the curve; CI, confidence interval; Sens, sensitivity; Spec, specificity; PPV, positive predictive value; NPV, negative predictive value; Brier, Brier score; FGVB, fetal growth velocity of the smaller twin; UA, umbilical artery. | | | | | | | |

Table S11A. XGBoost Hyperparameters

| **Parameter** | **Value** |
| --- | --- |
| nrounds | 8.00 |
| max_depth | 3.00 |
| eta | 0.01 |
| subsample | 0.80 |
| colsample_bytree | 0.80 |
| scale_pos_weight | 6.24 |
| min_child_weight | 3.00 |
| gamma | 0.10 |
| nfold | 5.00 |
| Hyperparameters selected via 5-fold cross-validation with early stopping (50 rounds). Fixed learning rate eta = 0.01. scale_pos_weight = (n_neg/n_pos) × 1.5 for aggressive imbalance handling. | |

Table S11B. XGBoost Model Performance Across Datasets

| **Dataset** | **N** | **AUC (95% CI)** | **Sens (%)** | **Spec (%)** | **PPV (%)** | **NPV (%)** | **Brier** |
| --- | --- | --- | --- | --- | --- | --- | --- |
| Training | 862 | 0.729 (0.686–0.773) | 64.1 | 72.5 | 35.9 | 89.4 | 0.136 |
| Test | 369 | 0.738 (0.670–0.806) | 81.0 | 60.5 | 29.7 | 93.9 | 0.126 |
| External | 699 | 0.710 (0.670–0.750) | 60.5 | 72.6 | 54.0 | 77.5 | 0.220 |
| Optimal threshold (Youden index from training set): 0.1850. Probabilities calibrated using Platt scaling with 5-fold CV predictions. AUC 95% CI calculated using DeLong method.  Abbreviations: AUC, area under the curve; CI, confidence interval; Sens, sensitivity; Spec, specificity; PPV, positive predictive value; NPV, negative predictive value. | | | | | | | |

## Table S12. Sensitivity Analyses of the Primary Association Model

| **Analysis** | **OR (95% CI)** | | | |
| --- | --- | --- | --- | --- |
|  | **Low rapid increasing** | **High decreasing** | **High increasing** | **FGVB** |
| Logistic model (Validation cohort) | 1.64 (0.89–3.00) | 1.95 (0.92–4.23) | 2.30 (0.34–45.95) | 0.62 (0.55–0.69) |
| Posterior probability-weighted model (Development cohort) | 1.66 (1.00–2.78) | 3.22 (1.86–5.55) | 2.92 (1.33–6.43) | 0.80 (0.72–0.88) |
| Center-fixed-effects model (Overall cohort) | 1.57 (1.08–2.27) | 2.67 (1.71–4.17) | 2.23 (1.13–4.44) | 0.72 (0.68–0.77) |
| **All models included discordance trajectory class (with Stable low as reference) and fetal growth velocity of the smaller twin (FGVB).** Posterior probability-weighted models were weighted by posterior probability of class membership. The center-fixed-effects model additionally included study center as a fixed covariate. | | | | |

##

## Table S13. Sensitivity Analysis Adjusting for Umbilical Artery Parameters (Development Cohort)

| **Variables** | **Univariate analysis** | | **Multivariable analysis** | |
| --- | --- | --- | --- | --- |
|  | **OR (95% CI)** | ***P* value** | **OR (95% CI)** | ***P* value** |
| Discordance trajectory class (reference: Stable low) |  |  |  |  |
| Low rapid increasing | 2.45 (1.54–3.83) | <0.001 | 1.57 (0.95–2.53) | 0.071 |
| High decreasing | 3.96 (2.29–6.74) | <0.001 | 2.46 (1.36–4.35) | 0.002 |
| High increasing | 6.76 (3.15–14.71) | <0.001 | 2.09 (0.83–5.20) | 0.112 |
| Fetal growth velocity - Smaller twin (per 10 g/week) | 0.74 (0.68–0.80) | <0.001 | 0.80 (0.73–0.87) | <0.001 |
| Abnormal UA Doppler | 6.65 (3.56–12.66) | <0.001 | 2.59 (1.22–5.50) | 0.013 |
| ﻿Intertwin UA PI discordance at last visit | 1.01 (1.00–1.02) | 0.046 | 1.00 (0.99–1.01) | 0.895 |
| Analyses were performed using the Development Cohort (Center = 1) with non-missing ﻿intertwin UA PI discordance (N = 1210). Intertwin UA PI discordance was defined as: (PI^larger^ − PI^smaller^) / PI^larger^ × 100%. Abbreviations: UA, umbilical artery; PI, pulsatility index. | | | | |

| **Table S14. Sensitivity Analysis Adjusting for Gestational Age at Delivery and Hypertensive Disorders of Pregnancy (Development Cohort)** | | | | |
| --- | --- | --- | --- | --- |
| **Variables** | **Univariate analysis** | | **Multivariable analysis** | |
|  | **OR (95% CI)** | ***P* value** | **OR (95% CI)** | ***P* value** |
| Discordance trajectory class (reference: Stable low) |  |  |  |  |
| Low rapid increasing | 2.46 (1.55–3.82) | <0.001 | 1.37 (0.79–2.32) | 0.254 |
| High decreasing | 4.42 (2.61–7.42) | <0.001 | 2.45 (1.32–4.47) | 0.004 |
| High increasing | 6.81 (3.55–13.22) | <0.001 | 1.00 (0.41–2.38) | 0.999 |
| Fetal growth velocity - Smaller twin (per 10 g/week) | 0.74 (0.69–0.80) | <0.001 | 0.94 (0.86–1.03) | 0.184 |
| Gestational age at delivery (weeks) | 0.45 (0.40–0.50) | <0.001 | 0.48 (0.42–0.54) | <0.001 |
| Hypertensive disorders of pregnancy | 2.25 (1.52–3.29) | <0.001 | 1.45 (0.92–2.26) | 0.106 |
|  | | | | |

| **Table S15A. Trajectory Model Comparison (Sensitivity Analysis: ≤32 weeks, ≥3 scans)** | | | | |
| --- | --- | --- | --- | --- |
| **Classes** | **BIC** | **Min class (%)** | **Entropy** | **APP** |
| 1 | 51,329.94 | - | - | - |
| 2 | 50,559.10 | 6.2 | 0.967 | 0.99 |
| 3 | 50,351.78 | 3.3 | 0.928 | 0.968 |
| **4** | **50,189.58** | **3.2** | **0.897** | **0.944** |
| 5 | 50,062.15 | 0.5 | 0.901 | 0.938 |
| Sensitivity analysis including only ultrasound examinations up to 32 weeks with at least 3 scans per pregnancy. N=982 in the development cohort; N=663 in the validation cohort.  Class proportions: 2-class: 93.8% / 6.2%; 3-class: 86.6% / 3.3% / 10.0%; 4-class: 3.2% / 85.1% / 4.9% / 6.9%; 5-class: 4.3% / 5.2% / 0.5% / 7.9% / 82.1%.  4-class model was selected as the optimal solution. 4-class model parameters: Class 1 (Stable low): intercept=7.11, slope=-0.015; Class 2 (Low rapid increasing): intercept=-7.90, slope=0.874; Class 3 (High decreasing): intercept=26.87, slope=-0.272; Class 4 (High increasing): intercept=12.18, slope=0.767.  Abbreviations: BIC, Bayesian Information Criterion; Min class (%), minimum class proportion; APP, average posterior probability. | | | | |

| **Table S15B. Logistic Regression for Composite Adverse Perinatal Outcomes (Sensitivity Analysis: ≤32 weeks, ≥3 scans)** | | |
| --- | --- | --- |
| **Variable** | **OR (95% CI)** | ***P* value** |
| Discordance trajectory class (reference: Stable low) |  |  |
| Low rapid increasing | 2.86 (1.65–4.87) | <0.001 |
| High decreasing | 4.49 (2.47–8.14) | <0.001 |
| High increasing | 4.95 (2.57–9.57) | <0.001 |
| *P* for trend |  | <0.001 |
| Fetal growth velocity - Smaller twin (per 10 g/week) | 0.95 (0.87–1.04) | 0.301 |
| Model adjusted for trajectory class and fetal growth velocity of the smaller twin (FGVB). | | |

| **Table S15C. Model Performance Metrics (Sensitivity Analysis: ≤32 weeks, ≥3 scans)** | | |
| --- | --- | --- |
| **Model** | **AUC Dev** | **AUC Val** |
| Base Model | 0.637 (0.590–0.683) | 0.632 (0.587–0.678) |
| Extended Model 1 | 0.638 (0.592–0.685) | 0.639 (0.594–0.684) |
| Extended Model 2 | 0.691 (0.648–0.733) | 0.646 (0.602–0.689) |
| Base Model = Trajectory class + FGVB; Extended Model 1 = Base Model + Abnormal UA Doppler; Extended Model 2 = Extended Model 1 + Chorionicity.  All models were fitted using the development dataset (Center 1). Validation metrics were calculated using the same models applied to the validation dataset (Center 2).  Abbreviations: Dev, development set; Val, validation set; Cal slope, calibration slope (ideal value = 1); FGVB, fetal growth velocity of the smaller twin; UA, umbilical artery. | | |

**Figure S1. Spaghetti plot (individual twin discordance trajectories by GBTM class). ﻿Colored lines: class−specific predicted trajectories; grey lines: individual patient trajectories.**


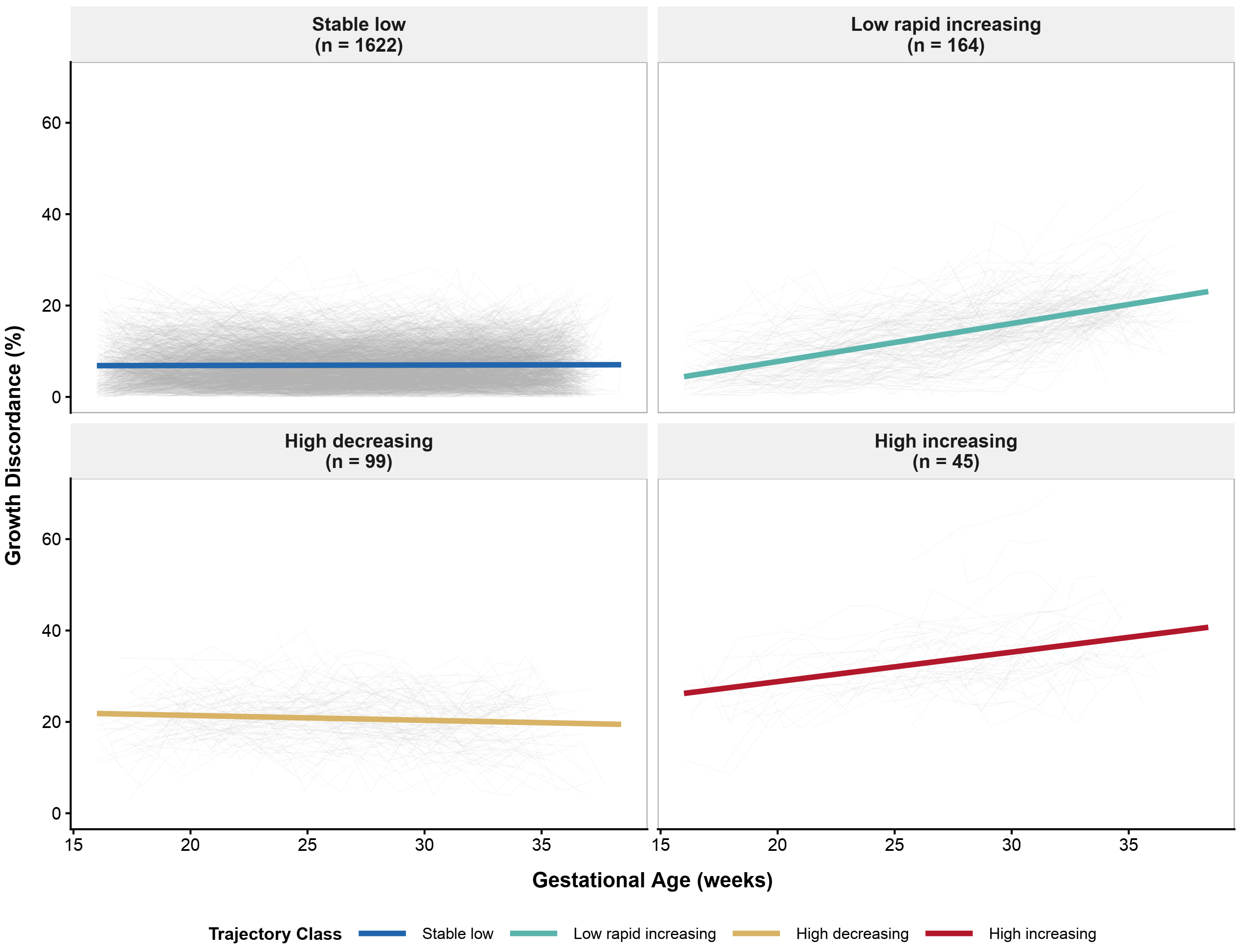


**Figure S2. Predicted Risk for Composite Neonatal Outcomes (Validation Cohort).** Predictions are from logistic regression model including discordance trajectory class, fetal growth velocity of smaller twin and their interaction terms.


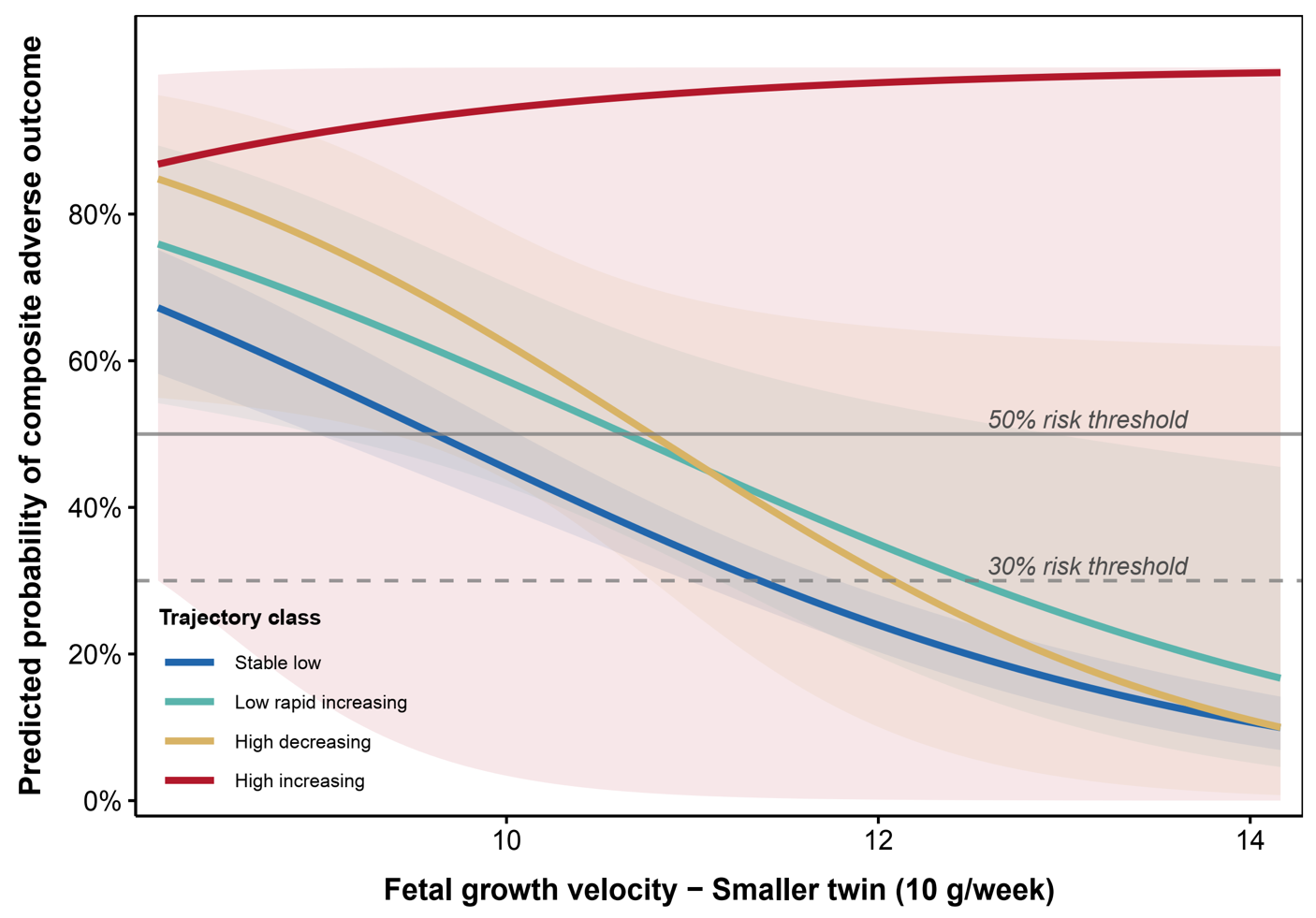


**Figure S3. SHAP Analysis for XGBoost Model Interpretation. (A) SHAP Summary Plot.**Summary of feature importance for the XGBoost model. SHAP values were calculated on the internal test set (Center 1, 30% hold-out), and mean |SHAP| values are shown. Color represents feature value. **(B) SHAP Dependence Plot for FGVB.**Non-linear relationship between fetal growth velocity of the smaller twin (FGVB) and predicted risk. SHAP values represent contribution to predicted risk (positive = increased risk). Color represents FGVB value. Dashed horizontal line at SHAP = 0 indicates no contribution.


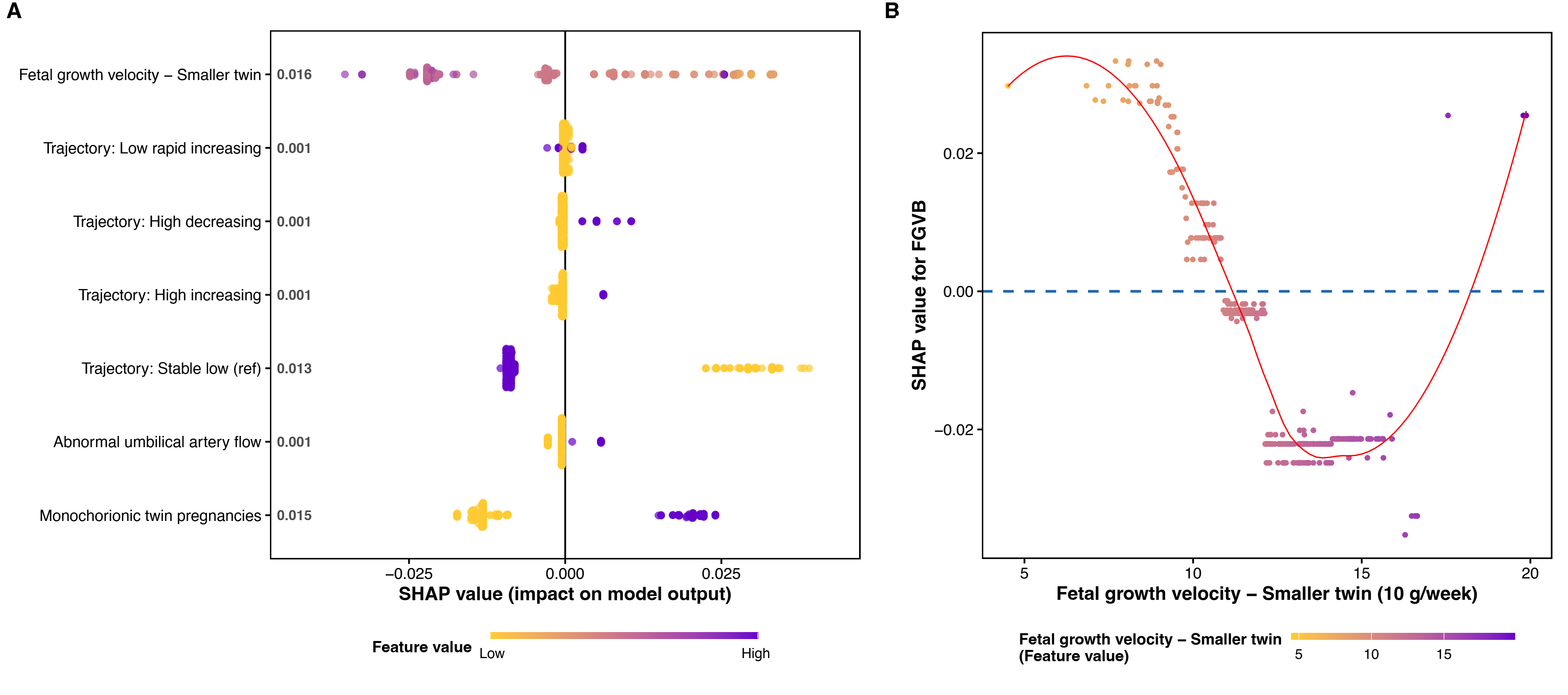

Supplement: Supplementary file 1 [file Data_sheet_1.docx]
